# Editorial: Application of innovative techniques in genetic and cellular therapies

**DOI:** 10.3389/fbioe.2023.1336900

**Published:** 2023-12-14

**Authors:** Yori Endo

**Affiliations:** Brigham and Women’s Hospital, Harvard Medical School, Boston, MA, United States

**Keywords:** cell therapy, gene therapy, exosome, miRNA, degenerative diseases

Cellular and gene therapies aim to restore or enhance cellular functionalities lost in disease or injuries. Cellular therapies, however are limited by both practical and legislative challenges. The current Research Topic covers some of the alternative avenues, such as the use of exosome as a surrogate for cellular autocrine/paracrine functions and how they may replace, supplement or even supplant cellular therapies in some aspects. It also explores various methods for improving targeted deliveries of drugs/nucleic acids beyond the use of viral vectors such as engineered exosomes and electroporation.

Cellular therapies have become one of the most explored avenues for treating degenerative diseases and injuries in the past few years. For some cases, cell transplantation is the only feasible alternative to tissue grafting or replacement, mitigation of which is desired for a number of reasons, including a requirement for invasive surgeries, donor site morbidities, and complications. Transplantation of cells allows harnessing of their functions, particularly that of regenerative functions. Their ability to respond to the environment at the transplant site through differentiation, release of factors etc., give them unique advantages during the whole course of regeneration (Sánchez et al., 2012; El-Kadiry et al., 2021). Poor cell survival, however, has been a major obstacle in achieving sufficient regenerative outcomes. In addition, the status of the cells at the time of transplantation seems to alter the cellular behavior post-transplantation and the subsequent clinical outcomes. Much of the recent innovation has therefore focused on the process of “priming.” In the context of priming, the induction of cellular quiescence has gained much attention due to its close relationship with the regenerative capacity of cells. Indeed, finding the appropriate method of inducing quiescence in progenitor cells has made a significant difference to the regenerative outcomes in skeletal muscle injuries (Xie et al., 2018; Endo et al., 2023). Similarly, Chen et al. described how quiescence preconditioning augmented the proliferation of the transplanted nucleus pulposus stem cells and improved the overall intervertebral disc regeneration. Priming, particularly the induction of quiescence, seems to be an innovative and effective method for maximizing the attainable regenerative functions in cellular therapies and therefore deserves continued research interest.

Exosome-based therapies are an attractive alternative to transplanting whole cells. Exosomes isolated from cells can perform some aspects of the complex paracrine/endocrine functions of the cells of origin, thus acting as their functional surrogates. The use of exosome has some potential commercial and clinical advantages, namely scalability, stability, (less) immunogenicity and accessibility to certain tissues across physiological barriers. In this Issue, Wang et al. reviewed recent findings showing that exosomes derived from different cell types found in bones perform unique functions by regulating gene expressions of the recipient cells, and suggested a potential therapeutic method based on selective utilization of such exosome for treating metabolic disorders of the bones.

Another innovative approach is to engineer exosomes as a vehicle for targeted delivery of various therapeutic agents, such as drugs and nucleic acids. One of the challenges that has prevented wider clinical applications of (natural) exosome has been their poor targeting ability. In this Issue, Jin et al. summarize some of the recently developed methods that allow exosomes delivery to specific targets that may otherwise be unreachable. Linking of exosomes with an anti-GAP43 monoclonal antibody or glycation end-products (RAGE)-binding-peptide allowed successful delivery of drugs or oligonucleotides to the brain in ischemic stroke treatment. The use of engineered exosomes may therefore become one of the optimal options in cases where tissue accessibility through physiological barriers and superior stability are necessary.

Similarly, the use of viral vector can achieve excellent topology and specificity for drug/nucleic acid deliveries. One exemplary such use explored in this Issue is for the delivery of miRNA inhibitors to regulate a group of gene expressions and subsequently the target cell activities. Wang et al. reported improved bone metabolism and slowed osteoporosis progression using adeno-associated virus expressing a miRNA-214 inhibitor. There is a strong collective evidence indicating efficacy of viral vectors. However, there remains a need for improved production efficiency for larger scale production in order to widen its clinical applications. Heuvel et al. argue that production of stable viral packaging cell suspensions holds the key to this. Their findings strongly indicate that the use of transposon-encoding mRNA is superior to plasmid-based transposase, and that this strategy should help establish virus VPCs producing vectors with a broadened host cell range. The use of virus as a vaccine platform is another innovative avenue explored and exploited in particular with an effort to combat COVID-19, including Alkayyal et al. The VSV∆51M oncolytic virus platform was repurposed to express the SARS-CoV-2 spike receptor-binding domain antigen, and successfully induced a humoral immune response in mice.

While others looked into developing biological vectors, there is a branch of research that is looking into physical methodologies to achieve localized drug/nucleic acid delivery. Electroporation is a method used for permeabilization of plasma membranes of biological cells, and its use is now is being explored for targeted drug/DNA delivery into living cells. Gene electro-transfer allows for delivery of DNA encoding therapeutic transgenes and its use is tested in cancer therapies and infectious diseases vaccines (Heller and Heller, 2015). While it is an attractive method with many practical advantages, Malyško-Ptašinskė et al. argue that establishment of optimal pulsing protocols is still necessary, and the treatment planning steps should include simulation of spatial electric field distribution and possible thermal effects.

In parallel with the development of delivery methods for drugs and nucleic acids, much effort has been made in discoveries of novel therapeutic targets for the treatment of degenerative conditions. The regulation of RNA modifications has attracted much research interest in the recent years as a potential therapeutic target for treating various conditions. In particular, m6A methylation has been extensively studied as it is the most common post-transcriptional modification of eukaryotic mRNAs and long non-coding RNAs. The exact roles of this particular RNA methylation in various physiological and pathological conditions seem complex, and a better understanding would most certainly benefit pioneering of a novel therapeutic avenue. In this Issue, Luo et al. and Wen et al. explored the roles of m6A methylation in wound healing and bone and intervertebral disc degeneration (IVDD), respectively. While both described that RNA methylation is a major regulatory pathway of cellular processes such as autophagy/apoptosis and proliferation, the effects reported are contradictory, highlighting the need for further research to establish viable therapeutic strategies. Aside from RNA modification, Wei et al., explained the roles of inflammatory and oxidative responses in IVDD pathogenesis and described the potential therapeutic values of anti-oxidant and anti-inflammatory therapies. While these avenues provide some promising prospects in developing novel therapies for IVDD, it was concluded that there has been too little evidence yet for comprehensive understanding of their therapeutic values. PI3K/AKT pathway regulates broad physiological and pathological processes, and Guo et al. suggest that its regulation may be an important factor in the development acute compartment syndrome that commonly occurs following severe fractures.
